# Assessment and characterization of back pain and biopsychosocial influences in lower limb amputation: scoping review

**DOI:** 10.1097/PR9.0000000000001504

**Published:** 2026-10-08

**Authors:** Kevin Arribart, Maximilian O'Malley, Gerson Cipriano, Marlon L. Wong, Robert S. Gailey, Ignacio A. Gaunaurd

**Affiliations:** a Department of Physical Therapy, University of Miami, Miller School of Medicine, Coral Gables, FL, USA; b Institut Merle d'Aubigné, Valenton, France; c Postgraduate Program in Health Sciences and Technologies, University of Brasilia, Brasilia, Brazil

**Keywords:** Amputation, Back pain, Measurements, Classification, Scoping review

## Abstract

Back pain affects over half of individuals with lower limb amputation, yet research findings on associated factors remain fragmented. In view of the evolution of pain science in the general population, this fragmentation likely stems from significant variability in the identification, characterization, and classification of pain, as well as inconsistent assessment of associated biopsychosocial factors. This scoping review aims to examine how back pain is currently identified, assessed, classified, and characterized in people with lower-limb amputation, to determine the gaps and inconsistencies that could impede standardization and evidence synthesis. Six electronic databases were searched for studies involving major unilateral acquired lower limb amputation. Two independent reviewers screened articles and extracted data using standardized forms in Covidence. Synthesis focused on identification, classification, reporting of back pain–related biopsychosocial variables, and assessment strategies. Sixty-seven studies were included, predominantly cross-sectional (69%). Although pain intensity was frequently assessed, methods were binary (yes/no), and the use of mechanistic classification and biopsychosocial factors was sparse. Measurement strategies were highly heterogeneous, with standardized frameworks used in only 4 studies. This scoping review reveals a profound discrepancy between contemporary pain science approaches and current pain practice methodologies for the lower limb amputation population. Significant diagnostic gaps persist because of inconsistent classification of pain mechanisms and a void in reporting biopsychosocial variables. Prioritizing standardized frameworks and a population-specific core outcome set is essential to bridge the translational gap. These advancements will improve research quality and facilitate more targeted, evidence-based clinical care for this complex population.

## Abstract 2

Supplemental Digital Content is Available in the Text.

Inconsistent pain classification and sparse biopsychosocial reporting across 67 studies reveal a translational gap, necessitating standardized frameworks to improve research quality for lower-limb amputation.

## 1. Introduction

Back pain is highly prevalent among people with lower limb amputation (LLA), with a majority (55%) reporting chronic back pain.^95^ This back pain is frequently characterized as intermittent and of moderate-to-severe intensity, typically persisting for over 1 hour daily.^32,95^ Under this broad clinical terminology, back pain can be partitioned along the spinal axis into distinct anatomical regions, each presenting independent epidemiological and biomechanical profiles. Specifically, low back pain (LBP), defined as extending from the 12th rib to the iliac crest,^66^ represents the most pervasive presentation, affecting approximately 71% of people with LLA globally, and between 53% and 72% when chronic.^95,134^ This is followed by sacral area symptoms, with chronic pain reported in 17% to 53% of the LLA population, and thoracic level pain, defined as pain experienced in the region of the thoracic spine,^107^ which concerns 10% to 32% of people with LLA presenting with chronic symptoms.^95^ Although impaired posture and limited functional mobility have often been considered primary etiologies of back pain for people with LLA,^33,43^ a variety of modifiable and nonmodifiable factors have been examined to determine their role as contributors to back pain in this population, including: (1) anthropometrics^3,6,20,32,34,68^; (2) socioeconomic factors^3,20,32,39,104,126^; (3) health status^3,6,11,20,32,34,38,39,75,101,104,115,116,118,126^; (4) psychological factors^20,83^; (5) biomechanical parameters^1,2,9,21,40,42,43,51–54,68,77,81,86,87,90,103,109,110,133^; (6) amputation characteristics^15,25,109,112,113,117^; and (7) prosthesis componentry.^45,60,105,125^ Although expansive, the results of these studies lack synthesis and consistency, making it difficult to establish clear relationships between these factors and back pain in people with LLA.

Over the past 15 years, the clinical understanding of how biopsychosocial factors influence back pain in the general population has evolved, leading to transformational shifts in assessment and treatment approaches.^28,66^ Concurrently, large-scale consensus initiatives have established standardized frameworks to optimize outcomes tracking; notably, the National Institutes of Health (NIH) Task Force introduced a core minimal dataset to standardize the collection of functional, sociocultural, and cognitive–affective domains,^35^ a practice further expanded by contemporary multidisciplinary consortia such as the NIH Back Pain Consortium Research Program.^82^ A pivotal development in this evolution was the 2016 introduction of “nociplastic pain” as a formal mechanistic descriptor, a concept that marked a paradigm shift in pain science and has since emerged as a critical focus in clinical literature.^67,93^ Despite these advancements, general knowledge and investigation into biopsychosocial factors and nociplastic pain mechanisms among people with LLA is scarce. This gap is clinically important given the profound physical, psychological, and social consequences inherent to major LLA.^4,111^

Investigating these contemporary factors and pain mechanisms is appropriate for the LLA population because these patients frequently navigate a multifaceted intersection of tissue trauma (nociceptive), nerve injury (neuropathic), and altered central processing. Recent conceptual work has sought to bridge this divide by adapting contemporary pain science frameworks to the LLA context, specifically describing the potential interplay between biomechanical and psychosocial factors.^41^

Lastly, the existing uncertainty regarding the relative contributions of modifiable and nonmodifiable factors to back pain is largely driven by several methodological limitations: (1) a lack of standardized assessment practices; (2) inconsistent classification of back pain by level, chronicity, and mechanism; or (3) inadequate reporting of biopsychosocial variables. These gaps may preclude robust meta-analyses and meaningful cross-study comparisons. Consequently, the literature concerning people with LLA may remain fragmented, exhibiting significant variability in how pain is identified, categorized, and treated, alongside inconsistent reporting of confounding factors, such as pain history, pain management strategies, and psychosocial status.^41,72,95^

Therefore, the following questions guided the development of this review for the population with LLA:

(1) How is back pain identified and classified (ie, terminology, location, pain status, chronicity, mechanism)?
(2) What associated biopsychosocial variables are commonly reported with back pain?
(3) What is the extent of methodological variability regarding the instruments and metrics used in the assessment of back pain and its associated variables?

A preliminary search of MEDLINE, the Cochrane Database of Systematic Reviews, and Joanna Briggs Institute Evidence Synthesis revealed no existing or ongoing systematic or scoping reviews on back pain reporting and assessment methodologies in people with LLA. A persistent lack of standardized assessment methodology, where factors such as phantom limb pain and secondary back pain are often inconsistently reported or excluded, may preclude robust data synthesis. The primary aim of this review was to examine how back pain is currently identified, assessed, classified, and characterized in people with LLA to determine the gaps that impede evidence synthesis. To address this aim, a scoping review design was selected rather than a systematic review. A scoping review is justified because the existing literature represents a highly heterogeneous body of evidence with poorly standardized clinical concepts and characteristics that preclude a narrow systematic synthesis or meta-analysis. By choosing this framework, it allows for mapping the breadth of current literature, clarifying evolving clinical concepts, and formally investigating research conduct in diverse study designs.^89^ By highlighting priority areas for improved standardization and mechanistic classification, this scoping review seeks to inform future research and clinical practice, providing the necessary foundation for targeted, evidence-based interventions tailored to the unique nuances of this complex patient population. To account for the high degree of terminological heterogeneity across the literature, “back pain” was adopted as a broad, inclusive term capturing low back, sacral, thoracic, and unspecified spinal pain.

## 2. Method

The scoping review protocol was drafted and conducted following the Joanna Briggs Institute and the extension for Scoping Reviews of the Preferred Reporting Items for Systematic Reviews and Meta-Analyses (PRISMA-ScR) guidelines.^100,123^ The final protocol was registered with the Open Science Framework on November 19, 2024 (https://osf.io/p4g5m).^74^

### 2.1. Search strategy

A comprehensive search strategy was developed in collaboration with a professional librarian, structured using the PCC framework (Population, Concept, Context) to ensure a systematic and reproducible approach. Utilizing keywords and index terms identified from relevant articles, 6 databases were investigated: PubMed, CINAHL, Cochrane Library, Web of Science, Scopus, and EMBASE (Supplemental Digital Content 1, https://links.lww.com/PR9/A451), followed by a manual screening of the reference lists of relevant literature for further relevant studies.

Studies that investigated major unilateral LLA, identified as transtibial, knee disarticulation, or transfemoral amputation levels, or their equivalents, were included. People with bilateral limb loss were excluded to maintain population homogeneity, as they exhibit distinct biomechanical gait adaptations, metabolic demands, and prosthetic clinical profiles compared with unilateral prosthesis users, which would introduce a significant confounding factor when mapping back pain characteristics. Included studies were required to assess the presence, characteristics, or related factors of back pain within their samples. The assessment of back pain could serve as either a primary or secondary outcome or as an inclusion criterion. No time restrictions were applied, and only published articles in English were considered for inclusion. Amputation of any acquired etiology was included, excluding those of congenital origin. Exploratory and explanatory study designs were included. Study protocols, conference abstracts, review articles, and case reports were excluded from the review.

All identified citations were imported into Covidence,^29^ where duplicates were removed. Two blinded, independent reviewers (K.A., M.O.) conducted a 2-stage screening process, first evaluating titles and abstracts, followed by a full-text review to determine final eligibility. Reasons for excluding articles at the full-text stage were documented. Any disagreements between reviewers were resolved by a third reviewer (I.G.). The complete selection process is illustrated using a PRISMA flow diagram,^96^ and inter-rater agreement (Cohen Kappa: κ) was calculated and interpreted for each screening stage following recommendations by Landis and Koch.^71^

Two reviewers (K.A., M.O.) independently extracted data using a standardized form in Covidence. Extracted study characteristics included bibliographic details (ie, authors, title, year, and journal of publication), location, study design, objectives, and inclusion and exclusion criteria. The population-specific demographic data included sample size, age, sex, amputation level and cause, time since amputation, comorbidities, mobility profile, level of activity, and prosthetic use.

Specific criteria were established to guide the choice of back pain-related variables extracted: (1) the variable served as a direct measure of back pain; (2) the variable was analyzed in direct relation to back pain; (3) the variable was used as a control in comparisons involving distinct pain states or back pain-related outcomes after an intervention. The selection and categorization of these variables were based on existing minimal datasets of demographic and outcome measures.^35,82^ In addition, we included specific clinical characteristics routinely evaluated during back pain assessment, alongside cognitive–affective and sociocultural concepts considered essential for both the amputation event and contemporary advancements in pain science since the production of these existing datasets.^24,66,119,130^ The selected variables are described in Table 1. When relevant, variables were divided into sub-categories presented in Table 1. The comprehensive tools used to characterize those variables were also reported.

**Table 1:** Back pain–related variables explored.

Variable	Subcategories
Aggravating factors	N/A
Alleviating factors	N/A
Classification	BP chronicity (eg, acute; recurrent; chronic); BP mechanisms (eg, nociceptive; neuropathic; nociplastic)
Comorbidities	Musculoskeletal; systemic; cognitive–affective (eg, depression, anxiety, post-traumatic stress disorder, catastrophizing pain, perceived injustice, apprehension, kinesiophobia)
Disability/interferences	Overall disability; bothersomeness; daily activity interferences; sleep interference; physical interferences; mood interferences; recreational, family, and social life interference; work ability
Duration of episodes	N/A
Frequency	Measurement method; occurrence setting
Identification	Pre-amputation; post-amputation
Intensity	Tool used; anchors; classification; occurrence setting
Location/extent	N/A
Management history	Pharmacologic; non-pharmacologic; surgical
Pain changes throughout time	N/A
Sensitivity/irritability	N/A
Sociocultural	Marital status/family support; social relationships/functioning; level of education; incomes; work status; work satisfaction; health access/environment (ie, health insurance status; area of living)
Terminology	N/A
Timeline	Time since first BP event; duration of BP episode; time since painful episodes

Variables without an identified subcategory were labelled.

BP, back pain; N/A: not applicable.

## 3. Results

The database search, conducted on December 13, 2024, followed by an update on March 19, 2026, yielded 16,927 citations, of which 5,823 were duplicates. In total, 11,104 records underwent title and abstract screening, resulting in 246 articles for full-text eligibility assessment. Three reports were unretrievable, and 2 additional studies were identified by reference screening. Of the 245 reports evaluated, 178 were excluded, leading to 67 studies included (Fig. 1). Inter-rater agreement was moderate during the initial screening (κ = 0.55), reflecting the deliberate breadth of the search strategy, and reached near-perfect consensus at the full-text stage (κ = 0.87) (Supplemental Digital Content 2, https://links.lww.com/PR9/A451).

**Figure 1.**
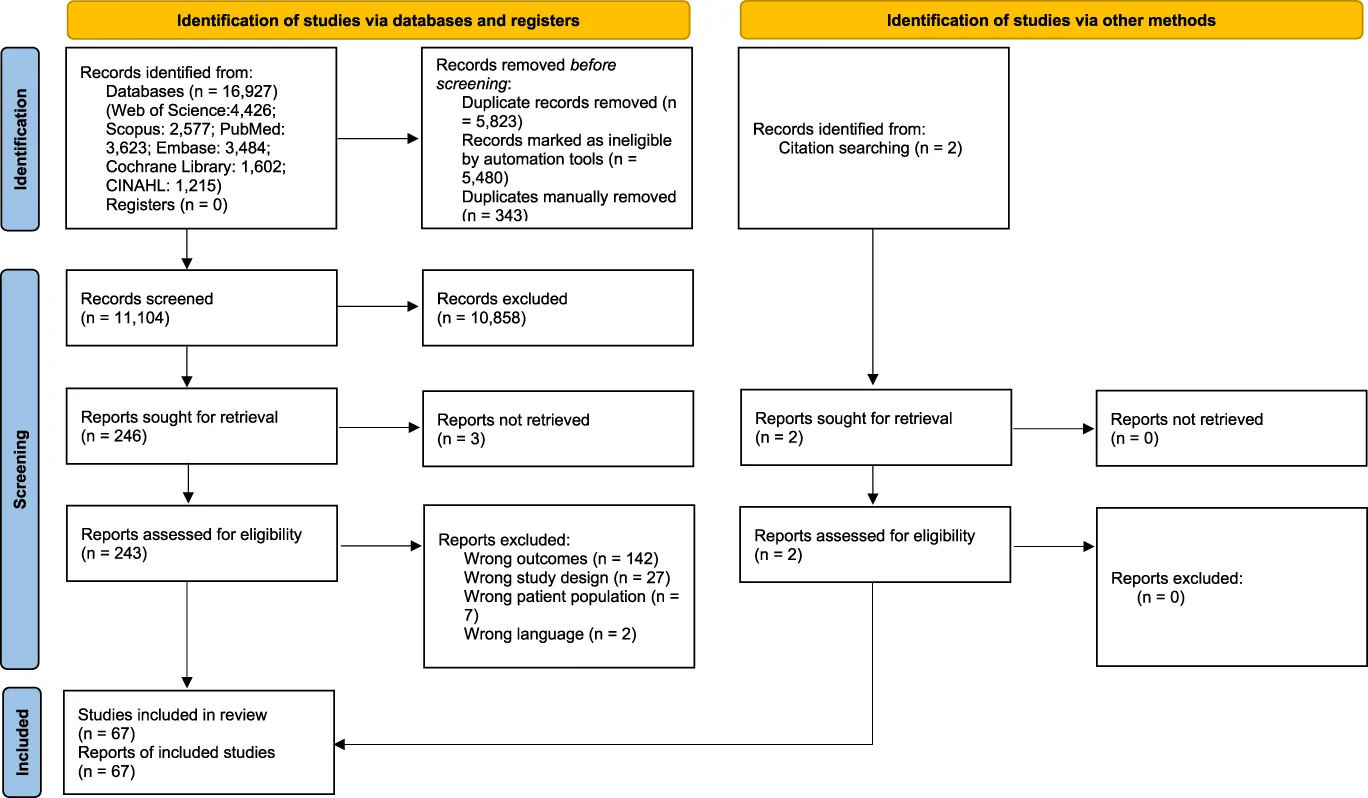
PRISMA flow diagram for new systematic reviews, which included searches of databases, registers, and other sources.^96^ PRISMA, Preferred Reporting Items for Systematic Reviews and Meta-Analyses.

### 3.1. Study characteristics

Of the 67 included studies, over half originated from the United States (54%, n = 36), with the remainder distributed across 17 countries, most notably Turkey (n = 5), New Zealand (n = 4), and Sweden (n = 3). Only 1 study predated 2000, 79% (n = 53) were published between 2010 and 2024. Cross-sectional designs predominated (69%, n = 46), whereas randomized controlled trials were sparse (n = 3) (Supplemental Digital Content 3, https://links.lww.com/PR9/A451). Most reports used nonspecific terminology, such as unspecified LBP (49%, n = 33) or unspecified “back pain” (30%, n = 20), while failing to clearly describe the precise anatomical boundaries of the pain or its chronicity within their cohorts. Chronicity, which is clinically defined as pain persisting or recurring for more than 3 months,^122^ was infrequently classified, with chronic LBP (cLBP) and chronic back pain identified in only 16% (n = 11) and 1% (n = 1) of studies, respectively. Detailed study location, year of publication, and study design are reported in Table 2.

**Table 2:** Characteristics of the included studies (N = 67).

Dimension	Count (%)
Location	
USA	36 (53.73)
Turkey	5 (7.46)
New Zealand	4 (5.97)
Sweden	3 (4.48)
Austria	2 (2.99)
Canada	2 (2.99)
Iran	2 (2.99)
The Netherlands	2 (2.99)
UK	2 (2.99)
Bosnia and Herzegovina	1 (1.49)
China	1 (1.49)
Estonia	1 (1.49)
France	1 (1.49)
Ireland	1 (1.49)
Italy	1 (1.49)
Korea	1 (1.49)
Palestine	1 (1.49)
Sri Lanka	1 (1.49)
Year of publication	
1990–1999	1 (1.49)
2000–2009	13 (19.40)
2010–2019	21 (31.34)
2020–2025	32 (47.76)
Study design	
Cross-sectional	46 (68.66)
Cohort	7 (10.45)
Quasi-experimental	6 (8.96)
Case–control	3 (4.48)
Randomized controlled trial	2 (2.99)
Case series	1 (1.49)
Crossover randomized controlled trial	1 (1.49)
Qualitative	1 (1.49)
Terminology used	
LBP	33 (49.25)
Back pain	20 (29.85)
cLBP	10 (14.93)
Acute LBP and recurrent LBP	1 (1.49)
Chronic back pain	1 (1.49)
Mechanical LBP	1 (1.49)
cLBP and LBP	1 (1.49)

cLBP, chronic low back pain; LBP, low back pain.

### 3.2. Back pain identification and classification

The identification of back pain presence was characterized by 12 distinct approaches used across 45 (67%) studies (Table 3). Binary self-report (eg, yes/no questions) was the most prevalent method (n = 15),^3,10,31,32,34,38,40,42,77,81,86,87,104,116,129^ followed by the interrogation of administrative health registries (n = 7),^18,19,58,75,83,84,132^ where researchers screened national medical databases using specific diagnostic codes (eg, International Classification of Diseases-10) to confirm the presence of back pain, and the descriptive report of pain frequency^39,50,73,103,118,126^ (Supplemental Digital Content 4, https://links.lww.com/PR9/A451). Notably, 16 studies assessed back pain status before amputation in addition to the status after amputation, although 7 of these used these data primarily as exclusion criteria.^20,23,44,62,81,86,87,132^

**Table 3:** Number of assessment strategies per variable and prevalence of reporting of the included studies, total and stratified by terminology used for localization and chronicity status (n = 67).

Variable	Strategies count	Articles count (n)						
		Total n (%)	LBP (n = 33)	Back pain (n = 20)	cLBP (n = 10)	Acute and recurrent LBP (n = 1)	Chronic back pain (n = 1)	cLBP and LBP (n = 1)
Aggravating factors	1	1 (1)			1			
Classification								
Chronicity	7	11 (16)	1		9	1		
Mechanisms	1	1 (1)	1					
Comorbidities								
Musculoskeletal	8	25 (37)	16	5	4			
Systemic	9	27 (40)	16	4	6			1
Affective	10	15 (22)	8	3	3	1		
Catastrophization	1	2 (3)			2			
Kinesiophobia	1	3 (4)	1		2			
Disability/interferences								
Overall disability	7	21 (31)	13	4	4			
Bothersomeness	5	7 (10)	1	5	1			
Daily activity interferences	4	7 (10)	2	4	1			
Sleep interference	3	2 (3)	2					
Recreational, family, and social life interference	2	3 (4)	1	2				
Work ability	2	3 (4)	1	2				
Duration of episodes	2	2 (3)		2				
Frequency								
Measurements	13	17 (25)	4	9	4			
Occurrence setting	6	14 (21)	3	7	4			
Identification								
Postamputation	12	45 (67)	23	15	4	1	1	1
Pre-amputation	4	16 (24)	10	3	1	1		1
Intensity								
Tool	9	46 (69)	23	14	9			
Anchors	13	30 (45)	18	8	4			
Classification	9	11 (16)	6	5				
Occurrence setting	14	32 (48)	16	9	7			
Management history								
Pharmacologic	5	7 (10)	5	1	1			
Nonpharmacologic	3	6 (9)	3	2	1			
Surgical	3	14 (21)	9	1	4			
Pain changes throughout time	1	1 (1)		1				
Sensitivity/irritability	1	1 (1)	1					
Sociocultural								
Marital status/family support	3	3 (4)	1	2				
Social relationships/functioning	2	3 (4)	1	2				
Level of education	5	5 (7)	2	2	1			
Incomes	1	1 (1)		1				
Work status	4	5 (7)	2	1	2			
Health access/environment	2	1 (1)		1				
Timeline								
Time since first BP painful event	2	5 (7)	2		3			
Time since it is been a problem without painful episodes	1	2 (3)	1		1			
Comprehensive tool	11	33 (49)	12	14	6		1	

LBP, low back pain; cLBP, chronic low back pain.

Among the 10 studies defining chronic back pain, definitions varied significantly regarding duration and frequency (Table 3). Although 4 studies adhered to a >3-month threshold,^30,32,108,127^ the remainder used 4 divergent criteria, ranging from intermittent symptoms over 12 months^31^ to specific combinations of persistence and symptom frequency (eg, pain on >50% of days over 6 months).^1,16,17,20^ One study was found to use a strategy to distinguish the neuropathic back pain mechanism.^3^

### 3.3. Reported variables in association with back pain

Pain intensity was the most frequently assessed variable (n = 46, 67%),^1,3,5–7,10,16–23,31,32,34,36,38–40,42,44,49,57,62,63,65,67,76,78–80,84–86,90,97,103,105,107,114,115,125–127^ yet reporting of metrics was inconsistent. Only 45% of studies defined measurement anchors,^5–7,10,16,22,31,32,34,36,38–40,42,44,62,63,77,79–81,86,87,91,98,104,106,115,126,127^ 16% used intensity-based group classifications,^22,34,38,39,44,63,68,104,115,126,127^ and 48% reported the occurrence setting for measurement (Table 3).^1,3,6,7,10,16–20,32,36,38,40,44,62,63,65,77,79–81,85–87,91,98,106,115,126–128^ Comorbidities were documented in 36 studies (54%), primarily focusing on musculoskeletal (37%)^3,5,6,16,18–20,23,32,34,39,40,42,55,62,65,68,70,81,86,87,91,104,121,126^ and systemic (40%)^3,5–7,18,20,23,27,30–32,34,39,42,58,62,65,68,70,75,81,86,87,91,104,108,114,126^ conditions, although affective comorbidities were less represented (22%).^6,19,20,32,39,50,80,81,83,85–87,104,121,127^ Catastrophization and kinesiophobia were reported in only 3%^20,80^ and 4%^7,20,80^ of the literature, respectively (Table 3).

Back pain frequency was reported in 25% of the studies,^1,17,20,32,34,36,39,50,68,73,79,103,104,115,116,126,127^ with 21% reporting information about the occurrence setting of the pain.^1,17,20,31,34,36,38,39,73,103,115,116,126,127^

Functional impact was predominantly captured through overall disability in 21 studies (31%),^5,6,16,20,22,31,40,44,45,50,57,63,69,70,79,81,85–87,108,127^ whereas specific life interferences (3%–10%),^10,31,34,36,38,116,127^ including bothersomeness (10%)^3,32,38,39,48,115,126^ and sleep (3%),^104,127^ were rarely addressed. Sociocultural factors reporting was sparse, with the most reported variable being the level of education^3,20,39,104,126^ and the employment status^3,20,32,126,127^(7% of the studies), followed by marital status^39,69,126^ and social relationships,^6,50,126^ in 4% of the studies. Incomes and health access or environment were reported in a single study,^39^ which reported household poverty status, insurance status, and the urban or rural setting. Management history was reported in 22 studies (33%),^3,5,18,19,23,27,30–32,34,42,44,50,57,62,68,70,104,108,115,127,132^ with 10% reporting pharmacological history,^23,27,31,62,115,127,132^ including the use of medication for back pain, 9% nonpharmacological history,^19,23,27,50,57,108^ and 21% the surgical management history^3,5,18,19,23,30–32,34,42,44,68,70,104^ (Supplemental Digital Content 4, https://links.lww.com/PR9/A451).

Clinical descriptors used for phenotyping, such as back pain onset,^1,17–20^ duration,^38,116^ time without painful episodes,^31,34^ aggravating factors,^32^ pain change,^116^ and sensitivity/irritability,^62^ were each limited or reported in a single report (1%–7%).

### 3.4. Reported assessment strategies for back pain and associated variables

Among 33 studies (49%) that used comprehensive assessment tools, author-developed questionnaires predominated (n = 17),^3,10,11,22,32,34,36,38,39,49,68,84,104,116,118,126,127^ whereas standardized frameworks like the NIH Minimal Dataset for cLBP were used in 4 studies (Supplemental Digital Content 4, https://links.lww.com/PR9/A451).^1,16,17,20^ Patient-Reported Outcome Measures designed specifically for the LLA population were used infrequently (n = 5),^48,103,115,126,128^ with 4 studies using the Prosthesis Evaluation Questionnaire or a modified version, Questions about Specific Bodily Sensations section with 3 of 16 questions related to back pain.^103,115,126,128^ One study used the Questionnaire for Persons with a Transfemoral Amputation, with 1 of 22 questions related to back pain.^48^

The assessment of pain intensity exhibited the highest degree of variability, primarily regarding temporal recall periods and events. Recall periods ranged from “current pain”^1,3,10,16,17,38,62,80,81,86,87,91,98,106^ to retrospective windows, from 1 week to 1 year.^1,6,7,20,32,36,38,40,44,65,79–81,85–87,115,126–128^ Recall events (ie, walking, use of a specific prosthetic foot) were used in only 2 studies.^63,77^

Considering instrumentation, the most used tool was a 0 to 10 numerical rating scale,^1,3,10,17,18,20,22,31,32,38,62,63,65,79–81,85–87,104,106,127^ used in 27 studies (40%), followed by a visual analogue scale, used in 14 studies (21%).^5–7,16,23,40,42,44,68,77,108,115,116,126^ Across studies, 9 different pain classifications were used to measure intensity (Supplemental Digital Content 4, https://links.lww.com/PR9/A451). Furthermore, among the studies that quantified intensity, 13 different semantic anchors for pain extremes (eg, “worst pain imaginable,” “pain as bad as could be”) were used.^5–7,10,16,22,31,32,34,36,38–40,42,44,62,63,77,79–81,86,87,91,98,104,106,115,126,127^ Pain frequency assessment followed a similar pattern of discordance, involving 13 distinct measurement strategies across 25% of studies (n = 17) (Supplemental Digital Content 4, https://links.lww.com/PR9/A451).^1,17,20,31,34,36,38,39,50,68,73,103,104,115,116,126,127^

Participant comorbidities were documented primarily as a method of determining study eligibility rather than classifying clinical phenotyping. Musculoskeletal and systemic conditions were used as exclusion criteria in 17 (25%)^5,16,18–20,23,40,42,62,65,68,70,81,86,87,91,121^ and 19 (28%)^5,6,18,20,23,30–32,34,42,62,65,70,81,86,87,91,108,114^ studies, respectively. When comorbidities were reported for pain classification, assessment relied on 8 different tools, ranging from the Charlson Comorbidity Index to simple binary checklists (Supplemental Digital Content 4, https://links.lww.com/PR9/A451). Affective dimensions were similarly fragmented, with 10 different strategies identified, including 3 (4%)^81,86,87^ considering this dimension as an exclusion criterion. Validated tools for pain-related cognition, such as the Pain Catastrophizing Scale and Tampa Scale of Kinesiophobia, were used in <4% of studies (Supplemental Digital Content 4, https://links.lww.com/PR9/A451).

Seven different measures and tools were used to describe disability presence and severity, most notably the Oswestry Disability Index (n = 7, 10%),^5,16,22,40,70,108,127^ its modified version,^20,44,45,69^ and the Roland-Morris Disability Questionnaire (n = 5, 7%) (Supplemental Digital Content 4, https://links.lww.com/PR9/A451).^31,50,81,86,87^ Assessments of “bothersomeness” and daily life interferences (sleep, social, or work-related) were assessed using 4 to 7 different measurement scales, respectively (Supplemental Digital Content 4, https://links.lww.com/PR9/A451).

Sociocultural factors and management history were markedly characterized by one-off or variable measurement strategies (Table 3).

All assessment strategies are available online in Supplemental Digital Content 4, https://links.lww.com/PR9/A451.

## 4. Discussion

Our first research question examined how back pain is currently identified and classified within the LLA literature. Notably, substantial variation was observed in how back pain was defined, classified, and assessed across studies. Current evidence suggests that the identification of back pain in this population predominantly relies on binary “yes/no” screening, which fails to capture the complexity of back pain. In terms of classification, the principal source of heterogeneity was inconsistent terminology. Nearly one-third of studies investigated broadly defined “back pain” without specifying anatomical location, whereas two-thirds investigated or evaluated LBP or cLBP specifically. None of the studies specifically investigated or evaluated the thoracic area for back pain in their population. This inconsistency limits comparison across studies and impedes adoption of standardized cLBP frameworks.^26,76,94,107^ Although pain duration thresholds are often appropriately tailored to specific study designs and research questions, the LLA literature is characterized by a significant heterogeneity in chronic back pain definitions. The chronic pain definitions range from 3 months to 1 year, with certain definitions incorporating symptom frequency requirements. Promoting more consistent application of standardized definitions may help enhance the comparability of future findings and facilitate more seamless evidence synthesis across the LLA population. To achieve standardized nomenclature, a formal consensus process, such as a Delphi study, involving an international, multidisciplinary panel of expert stakeholders is recommended to establish standardized terminology and core set of outcome measures suitable for this population. People with LLA may be predisposed to nociplastic pain.^1,16–18,20,30–32,56,75,80,102,108^ Yet, this review also found that mechanism-based assessment remains a novelty and is underused.^3,5^ To align with current pain science practice, future research may use validated frameworks like the Douleur Neuropathique 4 (DN4) or nociplastic criteria.^14,93^

Our second research question aimed to identify the biopsychosocial variables most commonly reported in association with back pain in the LLA population. A principle finding of this review is the systemic underreporting of biological, cognitive–affective, and sociocultural factors.^41^ Where other musculoskeletal conditions were reported, the classification was highly heterogeneous, ranging from spinal pathologies (eg, lumbar disc herniation, history of spinal trauma, spondylolisthesis)^5,18,20,62,70^ to residual^65,86,91^ or contralateral limb conditions.^40,42^ This inconsistent and limited consideration of musculoskeletal variables, specifically spinal pathologies and residual limb-related conditions, and systemic comorbidities highlights a substantial gap in the literature and underscores the need for standardized, validated approaches for comorbid assessment in people with LLA.

In addition, cognitive–affective factors were infrequently reported, with overall mental health and depression being the most reported variables, followed by anxiety.^6,19,20,31,32,39,50,81,83,85–87,104,121,127^ Post-traumatic stress disorder was reported in a single study,^83^ whereas catastrophization^20,80^ and kinesiophobia^7,20,80^ were reported in 2 and 3 studies, respectively. This finding is important because these 3 factors are components in recommended datasets or known to be influential in low back pain conditions in the general population.^35,82,120^ Perceived injustice, which is defined as a person's cognitive–affective appraisal of their illness or injury as unfair, can be characterized by perceptions of blame, a sense of unfairness, and irreparability of loss, and it has become a construct of interest in pain science between the late “90s and the early 2000s.”^13,124^ This scoping review did not find any studies that explored the role of perceived injustice on back pain in persons with LLA. Given the psychological adjustments and body-image-related modifications after amputation,^59,64^ and the role of perceived injustice in chronic pain,^99,131^ future research and clinical practice should explore this construct further in this population.

Only 15% of the studies^3,6,20,32,39,50,69,104,126,127^ reported on sociocultural factors that are present in minimal datasets or considered as associated with pain description in the general population.^35,82^ Although a single standardized measure for all sociocultural variables may not be feasible because of country-specific factors, a survey that captures marital status, work status, social relationships, education level, and area of living could be beneficial. Interestingly, no studies explored job satisfaction in relation to back pain in people with LLA, despite its potential role in the transition to chronic pain, and therefore its impact on employment.^130^ Therefore, reporting on the cognitive–affective and sociocultural factors must be improved to reflect their growing importance in pain science.^28^ The use of comprehensive tools, such as the NIH minimal dataset,^1,16,17,20^ which provides a global assessment of back pain including physical function and psychosocial factors,^35^ represents a significant opportunity for improved baseline and longitudinal data capture yet remains underused in this population.^82^ Augmenting these comprehensive tools with population-specific information (ie, residual limb, prosthetic use) can enhance their clinical and research adoption. Furthermore, it provides an opportunity to establish the reliability and validity of the augmented tool for people with LLA.

Furthermore, pain management history was predominantly documented as an exclusion criterion to mitigate potential confounding rather than as a descriptive variable for included participants.^5,18,23,30–32,34,42,44,50,57,62,68,70,104,108^ Beyond its application in study eligibility protocols, there was a near absence of detailed reporting regarding pharmacological,^27,115,127,132^ rehabilitative,^19^ and surgical management history.^3,19^ Given the established association between medication use, surgical history, and back pain in people with LLA,^97,115^ and the benefits of physical therapy interventions,^5,108^ consistent reporting is essential. Although excluding certain management histories may serve a specific internal validity purpose, it ultimately restricts the generalizability of findings and prevents a comprehensive understanding of the factors influencing back pain outcomes in this complex population.

Our third research question evaluated the extent of methodological variability regarding the instruments and metrics used to assess back pain and its associated variables. A significant opportunity exists to improve the reporting of the cognitive–affective and sociocultural factors, given their increasing consideration in pain science.^28^ The use of comprehensive tools, such as the NIH Task Force on Research Standards for Chronic Low Back Pain minimal dataset,^1,16,17,20^ which provides a global assessment of back pain, including physical function and psychosocial factors,^35^ may be adopted for better initial and longitudinal data capture, yet remains underused in this population.^82^ Notably, specific constructs such as perceived injustice and job satisfaction were absent from the reviewed literature. This omission is particularly salient given the profound body-image and social adjustments after major amputation,^59,64^ and the role of perceived injustice in chronic pain.^99,131^ Future research and current clinical practice should prioritize documenting and assessing the change over time of these constructs in relation to prosthetic rehabilitation and reintegration back to home and community activities. Similarly, despite the known interdependent relationship between job satisfaction, the emergence of chronic pain, and long-term vocational outcomes,^130^ no studies explored these factors in relation to back pain and people with LLA.

This scoping review identified a profound misalignment between modern pain science and current research practices within the LLA population. Our findings highlight 2 systemic issues: a lack of standardized classification practices and a pervasive void in the reporting of biopsychosocial variables. Specifically, the absence of mechanism-based classification, encompassing nociceptive, neuropathic, and nociplastic pain represents a significant diagnostic gap that limits meaningful clinical phenotyping.^67,92^ Although people with LLA may be uniquely predisposed to nociplastic pain because of the multifaceted intersection of tissue trauma, nerve injury, and altered central processing,^1,16–18,20,30–32,56,75,80,102,108^ this review found that mechanism-based assessment remains an underused novelty among recent included studies.^3,5^ To bridge this gap, future back pain research in people with LLA must prioritize the integration of validated frameworks, such as the DN4 questionnaire or established nociplastic criteria, to facilitate precise phenotype diagnosis and the development of targeted treatment strategies.^14,93^ In addition, the use of pain location descriptors, through body mapping, represents an opportunity to help onto the pain mechanism diagnosis,^78,93^ aiming to more targeted management.

The observed delay in adopting standardized assessments within the LLA literature reflects a broader “translational gap,” where research findings often fail to permeate routine practice.^8,61,88^ Despite the decades-long conceptual foundations of mechanism-based phenotyping, their integration into this population remains a “novelty” rather than a standard. This lag suggests that the shift toward mechanism-based assessment is not merely a matter of instrument availability but of innovation diffusion. Consequently, shifting from passive dissemination to active implementation science, restructuring research and clinical environments to make the use of “best evidence” the path of least resistance is essential to shorten this translational gap and improve outcomes for this complex cohort.^46^

This review revealed an important variability in assessment instrumentation. In addition to the variability in tools used, a barrier concerning the temporal recall periods and semantic anchors for pain intensity was highlighted. Comparing current back pain with 12-month retrospective back pain may lead to difficulties in interpreting results and understanding their impact on the factors explored. Moreover, it was noted that a lack of reporting of functional events (ie, standing, walking with the prosthesis) related to back pain occurrence was consistent across all studies. Future studies may consider incorporating evidence-based tools to ascertain mechanism-based back pain phenotyping in this population, such as the framework proposed by the Back Pain Phenotyping consortium's consensus recommendations for pain phenotyping.^93^ Furthermore, the proliferation of author-developed, nonvalidated questionnaires exacerbates the inability to assess and interpret disability in a standardized manner. Even when standardized tools like the Oswestry Disability Index or Roland-Morris Disability Questionnaire are used, their clinical utility can be questioned because of a lack of validation in the LLA population. To address these limitations without imposing an impractical burden on researchers, the focus may shift toward the development of a core outcome set specific to the LLA population. Rather than mandating standardized tools for every pain-related dimension, a prioritized framework targeting essential biopsychosocial domains would establish the common language required for longitudinal data capture and meta-analytical synthesis.

As discussed above, 21 of the included articles are using a broad “back pain” terminology without anatomical specifications, leading to a structural mismatch between available standardized frameworks and the terminology used in the LLA literature. Although comprehensive tools such as the NIH Task Force minimal dataset,^35^ the NIH Back Pain Consortium,^82^ and the Back Pain Phenotyping consortium's recommendations^93^ represent great opportunities for clinical reporting and phenotyping, these frameworks were explicitly developed and validated for LBP specifically rather than back pain globally. Given that spinal pain along the axial skeleton in people with LLA is heterogeneous, frequently encompassing independent thoracic and sacral area symptoms alongside lumbar presentations,^95^ applying LBP-specific tools to an undifferentiated “back pain” classification introduces substantial diagnostic noise. This mismatch further underscores the need to adopt precise, descriptive terminologies and clinical assessment instruments. Tailoring assessment strategies to the exact anatomical distribution of pain is an essential prerequisite for fostering high-fidelity research and implementing targeted, individualized therapeutic interventions in this complex patient population.

### 4.1. Strengths and limitations

This review provides a comprehensive overview of back pain assessment in people with unilateral LLA, spanning decades of research without time constraints. However, limitations include the exclusion of bilateral lower-limb and upper-limb loss populations, who possess distinct biomechanical and psychosocial profiles.^12,37,47^ The inclusion of all study designs, while deliberate to capture all assessment strategies, meant that many studies did not aim to consider back pain-related factors as controlling or exclusion criteria. The percentage of studies using each method was not reported to avoid exaggerating the lack of standardized practice. To ensure a comprehensive yet focused synthesis, outcomes were systematically extracted based on their methodological or conceptual relationship to back pain rather than a strict requirement for inferential statistical testing. This approach ensured that valuable descriptive data from small-sample studies were captured while avoiding the overinterpretation of variables completely unrelated to back pain management or characterization.

### 4.2. Implications for future research

Meaningful clinical and research progress in the LLA population who present with back pain may require greater standardization, shifting key practices from recommended to required. To this end, a 3-tiered assessment framework (standardizing back pain mechanism-based classification, back pain duration, and assessment of influential biopsychosocial factors) could improve the current methodological fragmentation. Widespread adoption of the NIH minimal dataset may provide a practical and feasible starting point; however, a core set of outcome measures validated for this population is still needed.

Building on this foundational clarity, the implementation of mechanism-based screening, specifically to distinguish between nociceptive, neuropathic, and nociplastic mechanisms, seems essential to transition from superficial description to precise clinical phenotyping. Ultimately, the integration of LLA-specific metrics into established biopsychosocial datasets will facilitate the development of a comprehensive outcomes set for the field. Such an integrated approach ensures that the unique biomechanical and prosthetic challenges of LLA are systematically captured alongside standardized pain metrics, providing a robust pathway for high-quality meta-analyses and evidence-based clinical guidelines.

This scoping review highlighted gaps in the literature and a need for comprehensive and standardized assessment methodologies for identifying, classifying, describing, and assessing back pain in people with LLA. Improvements in pain assessment practice may enhance the quality and generalizability of future research with this population, with the prospect of leading to more effective, targeted, and cost-efficient clinical care.

## Disclosures

The authors have no conflict of interest to declare. This research received no specific grant from any funding agency in the public, commercial, or not-for-profit sectors.

## Supplemental digital content

Supplemental digital content associated with this article can be found online at https://links.lww.com/PR9/A451.
