# Supplementary material for: Assessment and characterization of back pain and biopsychosocial influences in lower limb amputation: scoping review
Source: Pain Rep. 2026 Oct 8;11(6):e1504. doi: 10.1097/PR9.0000000000001504 (PMC13652451; doi:10.1097/PR9.0000000000001504)
Supplement: Supplementary file 1 [file painreports-11-e1504-s001.pdf]

Supplemental Digital Content 1: Search strategy

Keywords

**Amputation**	**Level of amputation**	**Back pain**
amputation	lower limb	back pain
amputee	lower extremity	backache
amputees	lower extrem*	backaches
amput*	leg	low back pain
limb loss	transtibial	lumbar pain
limb amputation	below-knee	lumbago
artificial limb	tibia	thoracic pain
artificial limb*	tibias	dorsalgia
prosthetic	tibiae	pain
prosthetically	knee	acute pain
prosthetics	through-knee	chronic pain
prosthese	knee disarticulation	sciatica
prosthesis	knee joint	spinal pain
prostheses	transfemoral	spinal disease
prosthe*	transfemorally	spinal disorder
Implants	above-knee	spinal dysfunction
prosthesized	thigh	spondylosis
	thighs	coccyx
	femur	coccydynia
	femure	
	femoral	

DATABASE	SEARCH STRATEGY	RESEARCH EQUATION
**PUBMED**	Filters:<br>ATM - Automatic Term Mapping (explosion)<br><br>Filters:<br>- Text availability: Abstracts<br>- Article language: English<br>- Species: Human<br>- Age: Adult	("amputation, surgical"[MeSH Terms] OR ("amputation"[All Fields] AND "surgical"[All Fields]) OR "surgical amputation"[All Fields] OR "amputation surgical"[All Fields] OR ("amputee s"[All Fields] OR "amputees"[MeSH Terms] OR "amputees"[All Fields] OR "amputee"[All Fields]) OR "amput*"[All Fields] OR "limb loss"[All Fields] OR "limb amputation"[All Fields] OR ("artificial limbs"[MeSH Terms] OR ("artificial"[All Fields] AND "limbs"[All Fields]) OR "artificial limbs"[All Fields]) OR "artificial limb*"[All Fields] OR ("prosthetic"[All Fields] OR "prosthetically"[All Fields] OR "prosthetics"[All Fields]) OR ("prosthetic"[All Fields] OR "prosthetically"[All Fields] OR "prosthetics"[All Fields]) OR ("prosthese"[All Fields] OR "prostheses and implants"[MeSH Terms] OR ("prostheses"[All Fields] AND "implants"[All Fields]) OR "prostheses and implants"[All Fields] OR "prostheses"[All Fields] OR "prosthesized"[All Fields]) OR ("prostheses and implants"[MeSH Terms] OR ("prostheses"[All Fields] AND "implants"[All Fields]) OR "prostheses and implants"[All Fields] OR "prosthesis"[All Fields]) OR "prosthe*"[All Fields]) AND ("lower extremity"[MeSH Terms] OR ("lower"[All Fields] AND "extremity"[All Fields]) OR "lower extremity"[All Fields] OR "lower limb"[All Fields] OR "lower extrem*"[All Fields] OR ("leg"[MeSH Terms] OR "leg"[All Fields]) OR "transtibial"[All Fields] OR "below-knee"[All Fields] OR ("tibia"[MeSH Terms] OR "tibia"[All Fields] OR "tibias"[All Fields] OR "tibia s"[All Fields] OR "tibiae"[All Fields]) OR ("knee joint"[MeSH Terms] OR ("knee"[All Fields] AND "joint"[All Fields]) OR "knee joint"[All Fields]) OR "through-knee"[All Fields] OR "knee disarticulation"[All Fields] OR ("transfemoral"[All Fields] OR "transfemorally"[All Fields]) OR "above-knee"[All Fields] OR ("thigh"[MeSH Terms] OR "thigh"[All Fields] OR "thighs"[All Fields] OR "thigh s"[All Fields]) OR ("femur"[MeSH Terms] OR "femur"[All Fields] OR "femurs"[All Fields] OR "femur s"[All Fields] OR "femoral"[All Fields] OR "femure"[All Fields])) AND

		("back pain"[MeSH Terms] OR ("back"[All Fields] AND "Pain"[All Fields]) OR "back pain"[All Fields] OR ("back pain"[MeSH Terms] OR ("back"[All Fields] AND "Pain"[All Fields]) OR "back pain"[All Fields] OR "backache"[All Fields] OR "backaches"[All Fields]) OR ("low back pain"[MeSH Terms] OR ("low"[All Fields] AND "back"[All Fields] AND "Pain"[All Fields]) OR "low back pain"[All Fields]) OR "lumbar pain"[All Fields] OR ("low back pain"[MeSH Terms] OR ("low"[All Fields] AND "back"[All Fields] AND "Pain"[All Fields]) OR "low back pain"[All Fields] OR "lumbago"[All Fields]) OR "thoracic pain"[All Fields] OR ("back pain"[MeSH Terms] OR ("back"[All Fields] AND "Pain"[All Fields]) OR "back pain"[All Fields] OR "dorsalgia"[All Fields]) OR "Pain"[MeSH Terms] OR ("acute pain"[MeSH Terms] OR ("acute"[All Fields] AND "Pain"[All Fields]) OR "acute pain"[All Fields]) OR ("chronic pain"[MeSH Terms] OR ("chronic"[All Fields] AND "Pain"[All Fields]) OR "chronic pain"[All Fields]) OR ("sciatica"[MeSH Terms] OR "sciatica"[All Fields] OR "sciaticas"[All Fields]) OR "spinal pain"[All Fields] OR "spinal disease"[All Fields] OR "spinal disorders"[All Fields] OR "spinal dysfunction"[All Fields] OR "spondylosis"[All Fields] OR "coccyx"[All Fields] OR "coccydynia"[All Fields])
**CINAHL WITH FULL TEXT (EBSCOHOST)**	Advanced search (Title/Abstract/Keyword)<br><br>Filters:<br>- Expanders: Apply equivalent subjects<br>- Source types: Academic journals<br>- Language: English<br>- Age: All adults	TI (("Amputation, Surgical" OR (Amputation AND Surgical) OR Amputees OR amputee OR amput* OR "limb loss" OR "limb amputation" OR (Artificial AND Limbs) OR "artificial limb*" OR prosthetic OR prosthetics OR prostheses OR prosthesis OR prosthe*) AND ((Lower AND Extremity) OR "Lower Extremity" OR "lower limb" OR "lower extrem*" OR Leg OR transtibial OR "below-knee" OR Tibia OR (Knee AND Joint) OR "Knee Joint" OR "through-knee" OR "knee disarticulation" OR transfemoral OR "above-knee" OR Thigh OR Femur) AND ((Back AND Pain) OR "Back Pain" OR backache* OR "Low Back Pain" OR "lumbar pain" OR lumbago OR "thoracic pain" OR dorsalia OR Pain OR "Acute Pain" OR "Chronic Pain" OR Sciatica OR "spinal pain" OR "spinal disease" OR "spinal disorders" OR "spinal dysfunction" OR "spondylosis" OR "coccyx" OR "coccydynia")) OR AB (("Amputation, Surgical" OR (Amputation AND Surgical) OR Amputees OR amputee OR amput* OR "limb loss" OR "limb amputation" OR (Artificial AND Limbs) OR "artificial limb*" OR prosthetic OR prosthetics OR prostheses OR prosthesis OR prosthe*) AND ((Lower AND Extremity) OR "Lower Extremity" OR "lower limb" OR "lower extrem*" OR Leg OR transtibial OR

		"below-knee" OR Tibia OR (Knee AND Joint) OR "Knee Joint" OR "through-knee" OR "knee disarticulation" OR transfemoral OR "above-knee" OR Thigh OR Femur) AND ((Back AND Pain) OR "Back Pain" OR backache* OR "Low Back Pain" OR "lumbar pain" OR lumbago OR "thoracic pain" OR dorsalia OR Pain OR "Acute Pain" OR "Chronic Pain" OR Sciatica OR "spinal pain" OR "spinal disease" OR "spinal disorders" OR "spinal dysfunction" OR "spondylosis" OR "coccyx" OR "coccydynia")) OR SU (("Amputation, Surgical" OR (Amputation AND Surgical) OR Amputees OR amputee OR amput* OR "limb loss" OR "limb amputation" OR (Artificial AND Limbs) OR "artificial limb*" OR prosthetic OR prosthetics OR prostheses OR prosthesis OR prosthe*) AND ((Lower AND Extremity) OR "Lower Extremity" OR "lower limb" OR "lower extrem*" OR Leg OR transtibial OR "below-knee" OR Tibia OR (Knee AND Joint) OR "Knee Joint" OR "through-knee" OR "knee disarticulation" OR transfemoral OR "above-knee" OR Thigh OR Femur) AND ((Back AND Pain) OR "Back Pain" OR backache* OR "Low Back Pain" OR "lumbar pain" OR lumbago OR "thoracic pain" OR dorsalia OR Pain OR "Acute Pain" OR "Chronic Pain" OR Sciatica OR "spinal pain" OR "spinal disease" OR "spinal disorders" OR "spinal dysfunction" OR "spondylosis" OR "coccyx" OR "coccydynia")) OR MW (("Amputation, Surgical" OR (Amputation AND Surgical) OR Amputees OR amputee OR amput* OR "limb loss" OR "limb amputation" OR (Artificial AND Limbs) OR "artificial limb*" OR prosthetic OR prosthetics OR prostheses OR prosthesis OR prosthe*) AND ((Lower AND Extremity) OR "Lower Extremity" OR "lower limb" OR "lower extrem*" OR Leg OR transtibial OR "below-knee" OR Tibia OR (Knee AND Joint) OR "Knee Joint" OR "through-knee" OR "knee disarticulation" OR transfemoral OR "above-knee" OR Thigh OR Femur) AND ((Back AND Pain) OR "Back Pain" OR backache* OR "Low Back Pain" OR "lumbar pain" OR lumbago OR "thoracic pain" OR dorsalia OR Pain OR "Acute Pain" OR "Chronic Pain" OR Sciatica OR "spinal pain" OR "spinal disease" OR "spinal disorders" OR "spinal dysfunction" OR "spondylosis" OR "coccyx" OR "coccydynia"))
**COCHRANE LIBRARY**	Advanced search (Title/Abstract/Keyword)	("Amputation, Surgical" OR (Amputation AND Surgical) OR Amputees OR amputee OR amput* OR "limb loss" OR "limb amputation" OR (Artificial AND Limbs) OR "artificial limb*" OR prosthetic OR prosthetics OR prostheses OR prosthesis OR prosthe*) AND ((Lower AND Extremity) OR "Lower Extremity" OR

		"lower limb" OR "lower extrem*" OR Leg OR transtibial OR "below-knee" OR Tibia OR (Knee AND Joint) OR "Knee Joint" OR "through-knee" OR "knee disarticulation" OR transfemoral OR "above-knee" OR Thigh OR Femur) AND ((Back AND Pain) OR "Back Pain" OR backache* OR "Low Back Pain" OR "lumbar pain" OR lumbago OR "thoracic pain" OR dorsalia OR Pain OR "Acute Pain" OR "Chronic Pain" OR Sciatica OR "spinal pain" OR "spinal disease" OR "spinal disorders" OR "spinal dysfunction" OR "spondylosis" OR "coccyx" OR "coccydynia") in Title Abstract Keyword
**WEB OF SCIENCE CORE COLLECTION**	TOPIC (Title/Abstract/Keyword)<br><br>Filters:<br>- Document types: Articles<br>- Languages: English	("Amputation, Surgical" OR (Amputation AND Surgical) OR Amputees OR amputee OR amput* OR "limb loss" OR "limb amputation" OR (Artificial AND Limbs) OR "artificial limb*" OR prosthetic OR prosthetics OR prostheses OR prosthesis OR prosthe*) AND ((Lower AND Extremity) OR "Lower Extremity" OR "lower limb" OR "lower extrem*" OR Leg OR transtibial OR "below-knee" OR Tibia OR (Knee AND Joint) OR "Knee Joint" OR "through-knee" OR "knee disarticulation" OR transfemoral OR "above-knee" OR Thigh OR Femur) AND ((Back AND Pain) OR "Back Pain" OR backache* OR "Low Back Pain" OR "lumbar pain" OR lumbago OR "thoracic pain" OR dorsalia OR Pain OR "Acute Pain" OR "Chronic Pain" OR Sciatica OR "spinal pain" OR "spinal disease" OR "spinal disorders" OR "spinal dysfunction" OR "spondylosis" OR "coccyx" OR "coccydynia") (Topic)
**SCOPUS**	(Title/Abstract/Keyword)<br><br>Filters :<br>- Document type: Article<br>- Language: English<br>- Keyword: Human<br>- Source type: Journal<br>- Publication stage: Final	TITLE-ABS-KEY (((amputation AND surgical) OR (amputees) OR amputee OR amput* OR "limb loss" OR "limb amputation" OR (artificial AND limbs) OR "artificial limb*" OR prosthetic OR prosthetics OR prostheses OR prosthesis OR prosthe*) AND ((lower AND extremity) OR "lower limb" OR "lower extrem*" OR leg OR transtibial OR "below-knee" OR tibia OR (knee AND joint) OR "through-knee" OR "knee disarticulation" OR transfemoral OR "above-knee" OR thigh OR femur) AND ((back AND pain) OR backache OR (low AND back AND pain) OR "lumbar pain" OR lumbago OR "thoracic pain" OR dorsalgia OR "Pain" [mesh] OR (acute AND pain) OR (chronic AND pain) OR sciatica OR "spinal pain" OR "spinal disease" OR "spinal disorders" OR "spinal dysfunction" OR "spondylosis" OR "coccyx" OR "coccydynia"))

**EMBASE**	Title/Abstract/Keyword<br><br>Filters:<br>- Age: Adult / Young adult / Middle aged / Aged / Very elderly<br>- Study type: Human<br>- Publication type: Article	(amputation,:ti,ab,kw AND surgical:ti,ab,kw OR amputees:ti,ab,kw OR amputee:ti,ab,kw OR amput*:ti,ab,kw OR 'limb loss':ti,ab,kw OR 'limb amputation':ti,ab,kw OR (artificial:ti,ab,kw AND limbs:ti,ab,kw) OR 'artificial limb*':ti,ab,kw OR prosthetic:ti,ab,kw OR prosthetics:ti,ab,kw OR prostheses:ti,ab,kw OR prosthesis:ti,ab,kw OR prosthe*:ti,ab,kw) AND (lower:ti,ab,kw AND extremity:ti,ab,kw OR 'lower limb':ti,ab,kw OR 'lower extrem*':ti,ab,kw OR leg:ti,ab,kw OR transtibial:ti,ab,kw OR 'below-knee':ti,ab,kw OR tibia:ti,ab,kw OR (knee:ti,ab,kw AND joint:ti,ab,kw) OR 'through-knee':ti,ab,kw OR 'knee disarticulation':ti,ab,kw OR transfemoral:ti,ab,kw OR 'above-knee':ti,ab,kw OR thigh:ti,ab,kw OR femur:ti,ab,kw) AND (back:ti,ab,kw AND pain:ti,ab,kw OR backache:ti,ab,kw OR (low:ti,ab,kw AND back:ti,ab,kw AND pain:ti,ab,kw) OR 'lumbar pain':ti,ab,kw OR lumbago:ti,ab,kw OR 'thoracic pain':ti,ab,kw OR dorsalgia:ti,ab,kw OR 'pain':ti,ab,kw OR (acute:ti,ab,kw AND pain:ti,ab,kw) OR (chronic:ti,ab,kw AND pain:ti,ab,kw) OR sciatica:ti,ab,kw OR 'spinal pain':ti,ab,kw OR 'spinal disease':ti,ab,kw OR 'spinal disorders':ti,ab,kw OR 'spinal dysfunction':ti,ab,kw OR 'spondylosis':ti,ab,kw OR 'coccyx':ti,ab,kw OR 'coccydynia':ti,ab,kw)

Supplemental Digital Content 2: Degree of agreement based on the title and abstract step selection (n = 11,104)

Reviewer A	Reviewer B	A Yes, B Yes	A Yes, B No	A No, B Yes	A No, B No	Proportionate Agreement	Yes Probability	No Probability	Random Agreement Probability	Cohen's Kappa
KA	MO	214	99	226	10,565	0.9707	0.0011	0.9333	0.9344	0.5537

Degree of agreement based on the full text review (n = 245)

Reviewer A	Reviewer B	A Include, B Include	A Include, B Exclude	A Exclude, B Include	A Exclude, B Exclude	Proportionate Agreement	Yes Probability	No Probability	Random Agreement Probability	Cohen's Kappa
KA	MO	65	3	10	167	0.9469	0.0850	0.5013	0.5863	0.8717

Supplemental Digital Content 3. Study design, sample size, classification method, and reported associated variables of the included studies (n = 67)

Author Year Location	Design	N	Terminology	Classification Method	Associated Variables
Acasio et al. 2022	Cross-sectional	50	cLBP	Duration and frequency	Frequency, intensity, pain timeline
Allami et al. 2019	Cross-sectional	247	LBP	Use of DN4	Bothersomeness, comorbidities (MSK, systemic), education, intensity, PMHx (surgical), pre/post amputation, work status
Anaforoğlu et al. 2016	RCT	40	Mechanical LBP	NR	Comorbidities (MSK, systemic), disability, intensity, PMHx (surgical), post amputation
Atar et al. 2022	Cross-sectional	42	LBP	NR	Comorbidities (MSK, systemic, affective), disability, intensity, social functioning
Atar et al. 2022	Cross-sectional	52	LBP	NR	Intensity, kinesiophobia
Behr et al. 2009	Cross-sectional	42	Back pain	NR	ADL interference, intensity, pre/post amputation
Beisheim et al. 2021	Cross-sectional	303	LBP	NR	Post-amputation
Butowicz et al. 2024	Cross-sectional	21	cLBP	Duration and frequency	Frequency, intensity, pain timeline
Butowicz et al. 2022	Cross-sectional	40	LBP	NR	Intensity, post-amputation
Butowicz et al. 2025	Case-control	30	cLBP	Duration	Comorbidities (MSK, systemic), intensity, pain timeline, PMHx (surgical), post-amputation
Butowicz et al. 2023	Case series	4	LBP	NR	Comorbidities (affective, MSK), intensity, pain timeline, PMHx (non-pharmacologic, surgical), pre/post-amputation

Butowicz et al. 2019	Cross-sectional	31	cLBP	Duration and frequency	Comorbidities (MSK), disability, intensity
Butowicz et al. 2020	Cross-sectional	33	cLBP	Duration and frequency	Catastrophization, comorbidities (affective, MSK, systemic), disability, education, frequency, intensity, kinesiophobia, pain timeline, pre-amputation, work status
Cacciola et al. 2024	Cross-sectional	57	LBP	NR	Disability, intensity, post-amputation
Çalışkan et al. 2024	RCT	29	LBP	NR	Comorbidities (MSK, systemic), intensity, PMHx (pharmacological, non-pharmacological, and surgical), pre-amputation
Clemens et al. 2025	Cross-sectional	135	LBP	NR	Comorbidities (systemic), PMHx (pharmacological, non-pharmacological), post-amputation
Devan et al. 2012	Cross-sectional	145	LBP	NR	Comorbidities (MSK, systemic), frequency, intensity, interferences (ADL), pain timeline, PMHx (surgical), post-amputation
Devan et al. 2015	Qualitative	11	cLBP	Duration	Comorbidities (systemic), PMHx (surgical), post-amputation
Devan et al. 2017	Cross-sectional	18	cLBP	Duration	Comorbidities (affective, systemic), disability, frequency, intensity, interferences (ADL), pain timeline, PMHx (pharmacological, surgical), post-amputation
Devan et al. 2017	Cross-sectional	208	cLBP	Duration	Aggravating factors, bothersomeness, comorbidities (affective, MSK, systemic), intensity, PMHx (surgical), post-amputation, work status
Döring et al. 2021	Cross-sectional	21	Back pain	NR	Frequency, intensity, interferences (ADL)

Ehde et al. 2001	Cross-sectional	255	Back pain	NR	Bothersomeness, duration of episodes, frequency, intensity, interferences (ADL, work), pre/post-amputation
Ephraim et al. 2005	Cross-sectional	914	Back pain	NR	Bothersomeness, comorbidities (affective, MSK, systemic), education, frequency, health environment, income, intensity, marital status, post-amputation
Facione et al. 2019	Cross-sectional	12	LBP	NR	Comorbidities (MSK), disability, intensity, post-amputation
Fatone et al. 2016	Cross-sectional	23	LBP	NR	Comorbidities (MSK, systemic), intensity, PMHx (surgical), post-amputation
Friel et al. 2005	Cross-sectional	19	LBP	NR	Disability, intensity, PMHx (surgical), pre-amputation
Gaffney et al. 2024	Cohort	14	LBP	NR	Disability
Hagberg et al. 2014	Case-control	39	Back pain	NR	Bothersomeness, post-amputation
Hahn et al. 2018	Cohort	70	Back pain	NR	Intensity
Hammarlund et al. 2011	Cross-sectional	46	Back pain	NR	Comorbidities (affective), disability, frequency, PMHx (non-pharmacologic), pre/post-amputation, social functioning
Heszlein-Lossius et al. 2018	Cross-sectional	254	Back pain	NR	Comorbidities (MSK), post-amputation
Hitzig et al. 2023	Cross-sectional	231	Back pain	NR	Disability, intensity, PMHx (non-pharmacological), post-amputation
Hogan et al. 2022	Cohort	44,028	LBP	NR	Comorbidities (systemic), post-amputation

Kablan et al. 2022	Quasi-Experimental	32	LBP	NR	Comorbidities (MSK, systemic), intensity, PMHx (pharmacological), pre-amputation, sensitivity
Kannenberg et al. 2022	Cross-sectional	46	LBP	NR	Disability, intensity
Klenow et al. 2024	Quasi-Experimental	26	LBP	NR	Comorbidities (MSK, systemic), intensity
Kulkarni et al. 2005	Cross-sectional	202 (40 clinical examination)	Back pain	NR	Comorbidities (MSK, systemic), frequency, intensity, PMHx (surgical), post-amputation
Kusljugić et al. 2006	Cross-sectional	37	LBP	NR	Disability, marital status
Lancere et al. 2023	Crossover RCT	13	LBP	NR	Comorbidities (MSK, systemic), disability, PMHx (surgical)
Leijendekkers et al. 2019	Cohort	40	Back pain	NR	Frequency, post-amputation,
Luetmer et al. 2019	Cohort	96	LBP	NR	Comorbidities (systemic), pre/post-amputation
Mahon et al. 2020	Cohort	32	LBP	NR	Intensity, post-amputation
Marshall et al. 2002	Cross-sectional	478	Back pain	NR	Disability, intensity
Mathis et al. 2020	Cross-sectional	40	cLBP	NR	Catastrophization, intensity, kinesiophobia
Matsumoto et al. 2019	Case-control	17	LBP	NR	Comorbidities (affective, MSK, systemic), disability, intensity, pre/post-amputation
Mazzone et al. 2020	Cross-sectional	79	Acute and Recurrent LBP	Duration	Comorbidities (affective), pre/post-amputation

Miller et al. 2001	Cross-sectional	435	Back pain	NR	Post-amputation
Morgan et al. 2017	Cross-sectional	1296	Back pain	NR	Comorbidities (affective), disability, intensity, post-amputation
Morgenroth et al. 2009	Cross-sectional	17	LBP	NR	Comorbidities (affective, MSK, systemic), disability, intensity, pre/post-amputation
Morgenroth et al. 2010	Cross-sectional	23	LBP	NR	Comorbidities (affective, MSK, systemic), disability, intensity, pre/post-amputation
Nia et al. 2022	Quasi-Experimental	8	Back pain	NR	Comorbidities (MSK, systemic), intensity, post-amputation
Parr et al. 2025	Cross-sectional	58	LBP	NR	Intensity, post-amputation
Reiber et al. 2010	Cross-sectional	581	Chronic back pain	NR	Post-amputation
Russell Esposito et al. 2014	Cross-sectional	28	LBP	NR	Frequency, post-amputation
Sadowski et al. 2022	Cross-sectional	161	LBP	NR	Comorbidities (affective, MSK, systemic), education, frequency, intensity, interferences (sleep), PMHx (surgical), pre/post-amputation
Seth et al. 2023	Cross-sectional	83	LBP	NR	Intensity, post-amputation
Shin et al. 2018	Quasi-Experimental	19	cLBP	Duration	Comorbidities (systemic), disability, intensity, PMHx (non-pharmacologic)
Sjödahl et al. 2001	Quasi-Experimental	9	LBP	NR	Comorbidities (systemic), post-amputation
Smith et al. 1999	Cross-sectional	92	Back pain	NR	Bothersomeness, frequency, intensity, PMHx (pharmacologic)

Smith et al. 2008	Cross-sectional	107	Back pain	NR	Duration of episodes, frequency, intensity, interferences (ADL, work), pain change, post-amputation
Stam et al. 2004	Cross-sectional	240	LBP	NR	Post-amputation
Taghipour et al. 2009	Cross-sectional	141	LBP	NR	Comorbidities (affective, MSK), post-amputation
Wang et al. 2022	Cross-sectional	66	Back pain	NR	Bothersomeness, comorbidities (MSK, systemic), education, frequency, intensity, marital status, post-amputation, social functioning, work status
Watson et al. 2025	Cross-sectional	1,143	LBP	Duration	Comorbidities (affective), disability, frequency, intensity, interferences (daily activities, recreational, family and social life, sleep, work ability), PMHx (pharmacologic), work status
Weber et al. 2022	Quasi-Experimental	20	Back pain	NR	Intensity
Wijekoon et al. 2023	Cross-sectional	170	Back pain	NR	Post-amputation
Yang et al. 2025	Cohort	16,068	LBP	NR	Post-amputation, pre-amputation, PMHx (pharmacologic)

ADL: activities of daily living; cLBP: chronic low back pain; DN4: Douleurs Neuropathiques 4; LBP: low back pain; MSK: musculoskeletal; NR, not reported; PMHx: Past Medical History; pre amputation = identification pre-amputation; post-amputation = identification post amputation; RCT: randomized controlled trial

Supplemental Digital Content 4: Back pain assessment strategies

Variable	Assessment strategy	N articles using the strategy	References
**Aggravating factors**			
	Question yes/no: sitting; standing; lifting; bending; walking; stair climbing; sit-to-stand; in and out a car	1	[32]
**Classification**			
**Chronicity**			
	cLBP: > 3 months	4	[30,32,109,128]
	cLBP: > 6 months, at least half of the days in the past 6 months	2	[1,17]
	cLBP: > 3 months and with pain on at least half the days in the past 6 months	2	[16,20]
	cLBP: recurrent or persistent > 3 months	1	[18]
	cLBP: intermittent 12 months	1	[31]
	Recurrent LBP: ≥ 2 LBP diagnoses at least 3 months apart	1	[84]
	Acute LBP: Does not meet recurrent criteria	1	[84]
**Mechanisms**			
	DN4	1	[3]
**Comorbidities**			
**Musculoskeletal**			
	Exclusion criteria	17	[5,16,18–20,23,40,42,62,65,69,71,82,87,88,92,122]
	Question yes/no	4	[3,32,34,105]
	Review of medical imaging by specialists	2	[18,69]
	Clinical examination	1	[6]
	Medical database	1	[19]
	Based on the frequency of pain in sites other than the back	1	[39]
	Number of comorbidities from the checklist	1	[55]

Self-report	1	[127]
**Systemic**		
Exclusion criteria	19	[5,6,18,20,23,30–32,34,42,62,65,71,82,87,88,92,109,115]
Charlson Comorbidity Index	4	[76,82,87,88]
Number of comorbidities from the checklist	3	[32,34,105]
Clinical examination	2	[6,69]
Self-report	2	[3,82]
Number of comorbidities from self-report	1	[39]
Number of comorbidities without precision about the collection	1	[127]
Specific-disorder related questionnaire	1	[27]
Medical database	1	[58]
**Affective dimensions**		
SF-36: Mental Health Scale	6	[6,50,82,87,88,122]
Exclusion criteria	3	[82,87,88]
CES-D	2	[39,84]
HADS	2	[20,31]
PCL-C	1	[84]
PHQ-9	1	[128]
Suggested sentences: Totally disagree - Partially disagree - Partially agree - Totally agree	1	[105]
PROMIS depression and anxiety score	1	[86]
Checklist	1	[32]
Medical database	1	[19]
**Catastrophization**		
PCS	2	[20,81]
**Kinesiophobia**		
TSK	3	[7,20,81]
**Disability/Interferences**		

**Overall disability**		
ODI	7	[5,16,22,40,71,109,128]
24-item RMDQ	5	[31,50,82,87,88]
mODI	4	[20,44,45,70]
PROMIS-PI	2	[63,86]
SF-36	1	[6]
SF-6D	1	[57]
Modified BPI	1	[80]
**Bothersomeness**		
NRS 0-10	2	[3,38]
VAS 0-100	2	[116,127]
Not at all bothered; slightly bothered; extremely bothered	1	[32]
3-point Likert scale, 0: not at all bothered; 1: somewhat bothered; 2: extremely bothered	1	[39]
5-point Likert scale, 0: no trouble; 1: slight trouble; 2: moderate trouble; 3: considerable trouble; 4: great deal of trouble	1	[48]
**Daily activity interferences**		
NRS 0-10	3	[10,38,117]
Question yes/no	2	[31,34]
NRS 1-10	1	[36]
EQ-5D-5L	1	[128]
**Sleep interference**		
Hours of sleep per night: Less than 4 hours; between 4 and 6 hours; between 6 and 8 hours; more than 8 hours	1	[105]
4-point Likert scale, 1: very satisfied to 4: very dissatisfied	1	[128]
5-point Likert scale, 1: not at all/none to extremely/very severe	1	[128]
**Recreational, family, and social life interference**		
NRS 0-10	2	[38,117]
EQ-5D-5L	1	[128]
**Work ability**		

	NRS 0-10	2	[38,117]
	EQ-5D-5L	1	[128]
**Duration of episodes**			
	A few minutes; several minutes to 1 hour; several hours; one day or longer	1	[38]
	10 min or less; 10 min to 1h; greater than 1 h but less than 1 day; greater than 1 day	1	[117]
**Frequency**			
**Measurements**			
	Every day or nearly every day in the past 6 months; At least half the days in the past 6 months; Less than half the days in the past 6 months	3	[1,17,20]
	On some days; on most days; every day	2	[31,34]
	Never; only once or twice; a few times (about once/week); fairly often (2-3 times/week); very often (4-6 times/week); several times every day; all the time or almost all the time	2	[104,127]
	Intermittent back pain; constant back pain with variations in intensity; constant back pain with little variation in intensity. If pain not constant: Once a week or less; 2–3 times/wk; 4–6 times/wk or more	1	[38]
	Occasionally (most weeks); Often (most days); Constant (nearly all the time)	1	[69]
	Never; sometimes; always	1	[39]
	No; yes, occasionally; yes, a few times a month; yes, several times a week; yes, daily	1	[50]
	No; yes, with episodes; yes, chronic (daily)	1	[74]
	Sometimes; frequently (more than one episode per week); constant (almost every day)	1	[105]
	Less than once weekly; 2–3 times weekly; 4–6 times weekly or greater than 6 times weekly	1	[117]
	VAS 0-100 from 0: never; 100: all the time	1	[116]
	NRS 0-10 from 0: none; 10: constant	1	[128]
	5-point scale: 0: never; 5: always	1	[36]

**Occurrence setting**		
Past 4 weeks	7	[31,34,38,39,104,116,127]
Past 6 months	3	[1,17,20]
Past week	1	[128]
Past month	1	[74]
Past 3 months	1	[117]
Past year	1	[36]
**Identification**		
**Post-amputation**		
Question yes/no	15	[3,10,31,32,34,38,40,42,78,82,87,88,105,117,130]
Medical database	7	[18,19,58,76,84,85,133]
Based on the frequency	6	[39,50,74,104,119,127]
Self-report without precision	4	[11,30,107,115]
Self-report through a checklist	3	[55,57,103]
Clinical Examination	2	[5,122]
Based on the intensity	2	[22,92]
Self-report through a pain map	2	[21,99]
Self-report through a semi-structured questionnaire	1	[69]
Self-report of history of LBP-related treatment	1	[27]
Based on the interference	1	[86]
Based on the bothersomeness	1	[48]
**Pre-amputation**		
Exclusion criteria	7	[20,23,44,62,82,87,88]
Question yes/no	4	[3,10,38,105]
Medical database	4	[19,76,84,133]
Based on the frequency	1	[50]
**Intensity**		
**Tool**		

	NRS 0-10	22	[1,3,10,17,18,20,22,31,32,38,62,63,65,80–82,86–88,105,107,128]
	VAS 0-10	9	[6,7,16,23,40,44,69,109,117]
	VAS 0-100	5	[5,42,78,116,127]
	0-10 scale (no precision)	3	[19,21,34]
	NRS 1-10	3	[36,39,99]
	Likert scale 0-10	1	[92]
	Likert scale: much better; better; no difference; worse; much worse	1	[49]
	Likert scale: 0: no impact; 1: mild impact; 2: moderate impact; 3: severe impact	1	[57]
	5-point ordinal scale	1	[129]
**Anchors**			
	No pain – Pain as bad as it could be	7	[10,34,38,80,82,87,88]
	No pain – Worst pain imaginable	5	[16,32,92,107,128]
	No pain – Most severe pain	3	[5,7,62]
	No pain – Severe pain	3	[44,78,81]
	No pain – Worst pain	2	[6,31]
	No pain – Most intense pain imaginable	2	[22,63]
	Extremely mild – Extremely intense pain	2	[116,127]
	Mild – Extremely intense pain	1	[39]
	No pain – Extremely strong pain	1	[36]
	No pain – Extreme pain	1	[42]
	No pain – Very intense pain	1	[40]
	No pain – Worst bearable pain you have experienced in your life	1	[105]
	Low – High	1	[99]
**Classification**			
	1-4: mild; 5-6: moderate; 7-10: severe	3	[34,38,39]
	1-3: mild; 4-6: moderate; ≥7: severe	2	[22,63]

0: no pain; 1-3: slight; 4-6: moderate; 7-9: severe; 10: worst bearable pain you ever experienced in your life	1	[105]
4-6: moderate; 7-8: severe; 9-10: extreme	1	[69]
0-33: mild; 34-66; moderate; 67-100: severe	1	[116]
67-100: mild; 34-66: moderate, 0-33: severe	1	[127]
>5: moderate to severe pain	1	[128]
Minimally painful: < 2 cm; Painful: > 2 cm	1	[44]
Minimally painful: < 5 cm; Painful: > 5 cm	1	[44]
**Occurrence setting**		
Current	14	[1,3,10,16,17,38,62,81,82,87,88,92,99,107]
Past 3 months	5	[38,80,82,87,88]
Past 4 weeks	4	[32,116,127,129]
Past week	4	[44,65,81,128]
Past 7 days	3	[1,20,86]
First diagnosis	2	[18,19]
Past month	2	[6,7]
Past 2 months	1	[40]
Past year	1	[36]
Most recent medical encounter	1	[19]
Worst	1	[3]
While walking	1	[78]
While using the current prosthetic foot	1	[63]
While using the previous prosthetic foot	1	[63]
**Management history**		
**Pharmacologic**		
Exclusion criteria	3	[23,31,62]
Non-prescription medication; prescription pain; Alcohol use; Street drugs	1	[116]
Self-report of previous medical treatment for LBP	1	[27]

	Question: “Have you ever taken an opioid medication? (eg, oramorph, MST, tramadol) Are you currently taking an opioid medication?”	1	[128]
	Medical database	1	[133]
**Non-pharmacologic**			
	Exclusion criteria	4	[23,50,57,109]
	Self-report about previous LBP-related treatment (i.e., doctor, chiropractor, physical therapist)	1	[27]
	Medical database	1	[19]
**Surgical**			
	Exclusion criteria	12	[5,18,23,30–32,34,42,44,69,71,105]
	Medical database	1	[19]
	Question yes/no	1	[3]
**Pain changes throughout time**			
	Intermittent; Constant, varying in intensity; Constant, not varying in intensity	1	[117]
**Sensitivity/Irritability**			
	Pressure pain threshold: paravertebral muscles with a digital algometer device at constant velocity and gradual pressure application (perpendicular to the site of the evaluation and approximately 1 kg/s)	1	[62]
**Sociocultural**			
**Marital status / Family support**			
	Married; divorced; widow; never married	1	[39]
	Married; single; widow(er)	1	[70]
	Single; married; divorced/widowed	1	[127]
**Social relationships / Functioning**			
	SF-36: social functioning	2	[6,50]
	Living dependence: Independent; dependent	1	[127]
**Level of education**			
	Primary Education; Secondary Education; Bachelor of Science; Master of Science; Ph.D	1	[105]

Illiterate/primary school; middle school; high school/college	1	[127]
Less than grade 12; high School graduate/GED; greater than grade 12	1	[39]
Primary/Secondary/High school; diploma; academic education	1	[3]
No high school diploma; High school graduate or GED; Some college with no degree; Occupational/technical/vocational program; Associate degree: academic program; Bachelor's degree; Master's degree; Doctoral degree; Unknown	1	[20]
**Incomes**		
Household poverty status: not poor; near poor; poor	1	[39]
**Work status**		
Question yes/no	2	[3,32]
Employed; unemployed	1	[127]
Working now; Looking for work, unemployed; Sick leave or maternity leave; Disabled due to back pain permanently or temporarily; Disabled for reasons other than back pain; Student; Temporarily laid off; Retired; Keeping house; Other; Unknown	1	[20]
Self-report followed by manual classification (i.e., Military or civilian full-time, part-time, student, athlete, full-time parent, unable to work due to illness, retired, other)	1	[128]
**Health access/environment**		
Uninsured; medicare; medicaid; private; other	1	[39]
Urban; rural	1	[39]
**Timeline**		
**Time since first back pain painful event**		
Less than 1 month; 1-3 months; 3-6 months; 6 months-1 year; 1-5 years; more than 5 years	3	[1,17,20]
Medical database	2	[18,19]
**Time since it's been a problem without painful episodes**		
Less than 3 months; 3 months or more but less than 7 months; 7 months or more but less than 3 years; 3 years or more	2	[31,34]
**Comprehensive tool**		

	Authors’ made questionnaire/survey	16	[3,10,11,22,32,34,36,38,39,49,69,85,105,117,119,127]
	NIH minimal dataset	4	[1,16,17,20]
	PEQ	3	[104,116,127]
	Chronic Pain Grade questionnaire	3	[82,87,88]
	Survey for prosthetic use	2	[55,103]
	2008 recommendations consensus for LBP	1	[31]
	Q-TFA	1	[48]
	EQ-5D-5L	1	[128]
	Self-reported medical questionnaire	1	[78]
	PROMIS-29 v1.0	1	[86]
	Modified PEQ	1	[129]

cLBP, chronic low back pain; DN4, Douleurs Neuropathiques 4; NRS, numerical rating scale; VAS, visual analog scale; ODI, oswestry disability index; RMDQ, roland-morris disability questionnaire; mODI, modified Oswestry disability index; PROMIS-PI, patient-reported outcomes measurement information system – pain interference; SF-36, short-form 36; SF-6D, short form 6 dimensions; BPI, brief pain inventory; HADS, hospital anxiety and depression scale; PCL-C, post-traumatic stress disorder checklist for civilians; CED-D, center for epidemiologic studies depression scale; PCS, pain catastrophizing scale; TSK, tampa scale of kinesiophobia; NIH, national institute of health; PEQ, prosthesis evaluation questionnaire; TAPES, trinity amputation and prosthesis experiences scales; Q-TFA, questionnaire for persons with a transfemoral amputation; PROMIS, patient-reported outcomes measurement information system; PHQ-9: Patient Health Questionnaire 9; EQ-5D-5L: EuroQoL 5-Dimension 5-Level
